# Comparison of the Effect of Ketamine, Ketamine-Midazolam and Ketamine-Propofol on Post-Tonsillectomy Agitation in Children

**DOI:** 10.21315/mjms2021.28.5.7

**Published:** 2021-10-26

**Authors:** Zeinabsadat Fattahi-Saravi, Reza Jouybar, Rezvan Haghighat, Naeimehossadat Asmarian

**Affiliations:** Anaesthesiology and Critical Care Research Centre, Shiraz University of Medical Sciences, Shiraz, Iran

**Keywords:** ketamine, propofol, agitation, tonsillectomy, pediatric

## Abstract

**Background:**

Emergence agitation (EA) in children is one of the most common complications following anaesthesia. We aimed to compare the effect of ketamine, ketamine-midazolam and ketamine-propofol on EA after tonsillectomy.

**Methods:**

This study was a randomised, double-blind clinical trial conducted on 162 children undergoing adenotonsillectomy surgery. The participants were randomly divided into three groups of receiving ketamine (0.5 mg/kg) (*N* = 54), ketamine (0.5 mg/kg) + propofol (1 mg/kg) (*N* = 54) and ketamine (0.5 mg/kg) + midazolam (0.01 mg/kg) (*N* = 54) 10 min before the end of the operation. At the time of the patients’ entry into the post-anaesthesia care unit (PACU) and at intervals of 5 min, 10 min and 20 min after that, consciousness, mobility, breathing, circulation and SpO_2_ were recorded. Modified Aldrete recovery score (MARS), the objective pain score (OPS) and Richmond agitation-sedation scale (RASS) were also evaluated.

**Results:**

At the time of entrance to the PACU and 5 min later, the ketamine-midazolam and ketamine-propofol groups had lower RASS scores than the ketamine group (*P* < 0.001); after 10 min and 20 min, the ketamine-propofol group showed the lowest RASS score (*P* < 0.001). Ketamine-propofol group had a significantly lower MARS score at all-time points (*P* < 0.001). Recovery time was the longest for the ketamine-propofol group (*P* = 0.008).

**Conclusion:**

The ketamine-midazolam group had lower RASS, greater haemodynamic stability and MARS values without delayed awakening.

## Introduction

Emergence agitation (EA) in children is one of the most common early complications after anaesthesia, manifesting with behavioral symptoms such as restlessness, disorientation, excitation, impulsive movements and crying. EA increases the stress and workload of nurses and patients’ families, makes it difficult to monitor and care for the patients, delays discharge from recovery (post-anaesthesia care unit [PACU]) and can lead to physical injury. On the other hand, EA triggers sympathetic system activation, consequently increasing the heart rate and blood pressure and giving rise to complications such as wound bleeding; it also contributes to brain and heart damage (1–3).

The prevalence of EA varies from 0.25%–90.5% (4). Various factors play a role in the occurrence of EA, such as post-operative pain, reduced effect of anaesthesia drugs in recovery, type of drug and anaesthesia technique, type and location of surgery, age, stress during induction of anaesthesia, hypoxemia, airway obstruction, environmental noise, duration of anaesthesia, rapid awakening in an unfamiliar environment, sore throat, bladder distention, patient personality, and the presence of a tracheal tube, obesity, cognitive impairment, psychiatric problems, nasogastric tube or urinary catheter (5–9). One of the most common surgical procedures in children is adenotonsillectomy, which is associated with post-operative pain and bleeding. EA is very common in otolaryngology procedures, increasing the rate of post-operative airway obstruction and its complications.

In previous studies, various drugs have been used to prevent EA and reduce its symptoms (10–15). Ketamin-propofol is one drug that has been effective in reducing the rate of EA following tonsillectomy. Other drugs used to prevent EA include fentanyl, dexmedetomidine, sufentanil, midazolam, clonidine, alfentanil and remifentanil (10–15). Propofol, which is one of the drugs used for induction and maintenance of anaesthesia, can also prevent EA via various mechanisms (16–17).

Midazolam is a short and fast acting benzodiazepine that insert its effect through the ionotropic GABA (A) receptors in the central nervous system. Midazolam has anxiolytic, sedative, hypnotic, anterograde amnesia, muscle relaxation and anti-convulsion properties. Although benzodiazepines do not have any analgesic effect, they have synergistic interactions with hypnotic and opioids. In previous study combination of ketamine-midazolam was used for post-operation agitation (18–19).

To the best of our knowledge, in previous studies, the effectiveness of ketamine, ketamine-propofol and ketamin-midazolam on EA following tonsillectomy has not been compared, comprising the subject of the present investigation. Therefore, we aimed to finding the best modality of controlling post-tonsillectomy agitation in children, decreased PACU duration by control post-operation agitation and decreased post-operative complications.

## Methods

This study was a randomised, double-blind clinical trial conducted on 150 children undergoing adenotonsillectomy surgery at the Khalili and Dastgheib hospitals affiliated to the Shiraz University of Medical Sciences (Shiraz, Iran) from June to November 2018. The study protocol was in accordance with the Declaration of Helsinki and Good Clinical Practice guidelines and was approved by the Ethics Committee of Shiraz University of Medical Sciences. This study was registered in the Iranian Registry of Clinical Trial, where the trial protocol could be accessed. Written informed consent was obtained from the guardians of the eligible participants.

The inclusion criteria were all children aged 5 years old–15 years old with the American Society of Anesthesiologists (ASA) class I–II undergoing adenotonsillectomy surgery, while the exclusion criteria included patients with neurologic disorders, congenital heart defect, upper respiratory tract infection, convulsion, drug reaction/hypersensitivity, history of post-operative complications, intraoperative complications or need for reoperation due to bleeding.

After entering the operation room, the patients were placed in one of the groups by the nurse who was not involved in this study. Then, 10 min before the end of the operation, the drug was injected by the nurse who was not aware of the study procedure. The patients were divided into three groups of receiving ketamine (0.5 mg/kg) (group 1, *N* = 54), ketamine (0.5 mg/kg) + propofol (1 mg/kg) (group 2, *N* = 54) and ketamine (0.5 mg/kg) + midazolam (0.01 mg/kg) (group 3, *N* = 54). Randomisation of the study was performed for 162 participants using 27 blocks with size of 6 from *http://randomization.org/*and patients were divided into three equal groups.

Patients, physicians and research staff were blind to this study. Patients and the investigators were unaware of the types of drugs. To blind investigators, all the medication prepared by an anaesthesiologist was numbered similarly, and the medication was given to a nurse and then the nurse gave it to the anaesthesiologist who performed the procedure. Also, generating the random allocation sequence, measurements, assigning participants to interventions were done by individuals who were blinded to the study.

All patients after entering the operating room were monitored by electrocardiogram (ECG), pulse oximetry and non-invasive blood pressure (NIBP) monitoring. Then, the intravenous catheter was implanted in the upper limbs to hydrate the patients with Ringer’s lactate (6 mL/kg). Induction of anaesthesia was performed with propofol (2 mg/kg–3 mg/kg) and atracurium (0.5 mg/kg) as a sedative, and fentanyl (23 μg/kg) was used for analgesia during the surgery. Maintenance of anaesthesia was established with isoflurane (1.2 minimum alveolar concentration [MAC]–1.8 MAC), N_2_O and O_2_, and end-tidal CO_2_ (between 30 mmHg and 35 mmHg).

All patients were injected with ondansetron (0.15 mg/kg) and dexamethasone (0.2 mg) after induction of anaesthesia to prevent post-operative nausea and vomiting. After the operation, isoflurane and nitrous oxide were discontinued and the residual muscle relaxant effect was reversed by neostigmine (0.06 mg/kg) and atropine (0.02 mg/kg). After extubation and spontaneous respiration, each patient was transferred to the PACU with a respiratory rate of > 12/min, tidal volume > 8 mL/kg and SpO_2_ > 98%, where they were monitored in terms of heart rate, mean arterial blood pressure (MABP) and SpO_2_. An O_2_ facial mask was installed if needed.

At the initial time of the patients’ entry into the PACU and then at intervals of 5 min, 10 min and 20 min, consciousness, mobility, breathing, circulation and SPO_2_ were recorded for all patients with the modified Aldrete recovery score (MARS) (Table 1).

MARS is used to determine the time that patient is ready for discharge and compromises five criteria including motor activity, respiration, blood pressure, consciousness and colour. Each item scores 0–2 and overall score would be 0–10. Scores closer to 0 indicate that the patient is closest to the anaesthesia state. Scores of 9 and above indicate that the patient can be discharged (20).

Furthermore, pain was evaluated using the objective pain score (OPS) which objectively demonstrates the requirement of analgesia in patients with mild-to-moderate pain. It scores 1–4. Inadequate pain relief/pain at rest is equivalent to score 1, pain free at rest/normal breathing = 2, pain free when deep breathing/incentive spirometry, but pain when coughing = 3 and pain free when deep breathing/incentive spirometry, but pain when coughing = 4 (21).

Agitation was measured using the Richmond agitation-sedation scale (RASS) (Table 1). RASS evaluate both agitation and sedation in adult and responsiveness in children. It is a 10-point scale ranging from −5 to 4. Levels −1 to −5 denote 5 levels of sedation, starting with ‘awakens to voice’ and ending with ‘unarousable.’ Levels +1 to +4 describe increasing levels of agitation. The lowest level of agitation starts with apprehension and anxiety, and peaks at combative and violent. RASS level 0 is ‘alert and calm’ (22).

All evaluations were made by an independent nurse. If the patients’ pain was more than 4 (OPS ≥ 4), diclofenac (1 mg/kg) was administered in the form of a suppository.

The sample size was calculated based on the 2016 Kim et al. (23) study. Each group required at least 34 patients assuming a 30% difference between the groups in the incidence of EA, a type I error rate of 5%, 80% power and a dropout rate of 10%.

## Data Analysis

In this study, data had abnormal distribution with Kolmogrove-Smirnove test (*P*-value < 0.05), therefore, continuous variables were reported as the median and interquartile range (IQR). The Kruskal-Wallis test with Dunn’s test with Bonferroni correction as post-hoc test was used to compare the medians among the three groups. Categorical variables were reported as a number and percentage, with the chi-squared test being used to compare proportions between groups. Since the interaction between time and group was significant, we could not report the overall effect over time. Thus, we reported the differences at the individual time points between the groups. Data were analysed using SPSS 21 and *P*-values < 0.05 were considered statistically significant.

## Results

**Figure 1:**
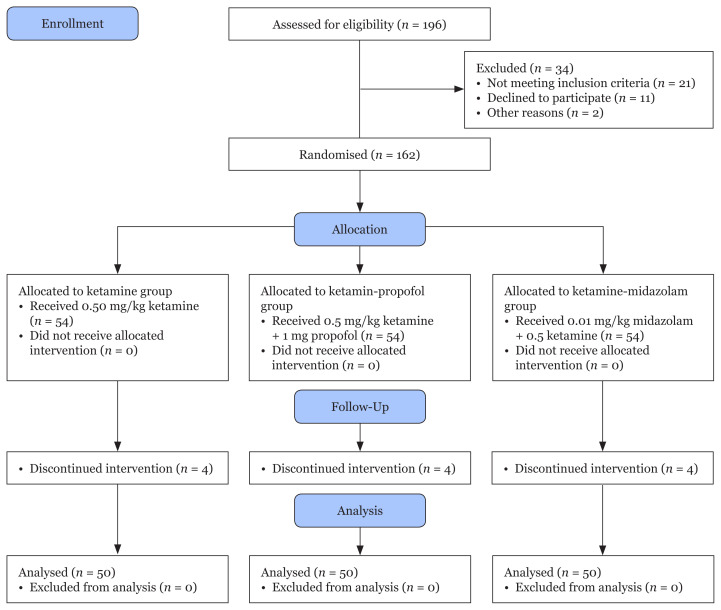
Consort flow diagram

Among 196 eligible patients enrolled in the study, 34 were excluded as they did not meet the inclusion criteria or declined to participate. A total of 162 patients scheduled to undergo adenotonsillectomy in our hospital between June and November 2018 participated, 150 of whom completed the study (Figure 1). The demographic characteristics of the three groups of children are shown in Table 1.

Table 2 shows the median (IQR) of the RASS, MARS and OPS indices during the study. The scores of these scales varied significantly among the groups at different time points (*P* < 0.001). Based on the post-hoc test, a difference in RASS was found among all groups at baseline and 5 min after surgery; furthermore, the ketamine-propofol group varied significantly in this index from the other two groups 10 min and 20 min after surgery.

In the MARS, the ketamine-propofol group had a significant difference with the other two groups at all time points (*P* < 0.001). In the OPS, there was a difference at baseline and 20 min after surgery between the ketamine group and the other two groups, and among all groups at 5 min and 10 min after surgery (*P* < 0.001). Recovery time was the longest for the ketamine-propofol group (*P* = 0.008).

## Discussion

Emergence agitation (EA) is one of the common complications in PACUs, occurring frequently in children (24). This can have negative impacts on the patient and caregivers. Therefore, various drugs and methods have been used to reduce agitation. In this study, the efficacy of ketamine-midazolam was compared with that of ketamine-propofol and a ketamine (control) in reducing EA. Our results showed that giving the above two compounds reduces the RASS compared to the ketamin group. At the time of entrance to the PACU and 5 min later, the ketamine-midazolam and ketamine-propofol groups had lower RASS values than the ketamin group (*P* < 0.001); after 10 min and 20 min, the ketamine-propofol group showed the lowest RASS (*P* < 0.001). On the other hand, the MARS was lower in the ketamine-propofol group than the other two groups at all time points (*P* < 0.001).

In the study of Jalili et al. (16), patients received an infusion of ketamin-propofol (intervention group) or ketamine (control group) during the surgery. Their results revealed that the rate of EA was worse in the ketamin-propofol group than in the ketamin group; however, this was not significant.

In another study, at the end of the tonsillectomy operation, ketamin-propofol and propofol were given in isolation. The rate of EA, pain and haemodynamic stability was better in the ketamin-propofol group than the propofol group (25). The above findings regarding post-operative agitation in the ketamin-propofol group are consistent with our findings. However, in our study, patients in the ketamine-midazolam group had less agitation than the ketamin group when entering the PACU and 5 min later (*P* < 0.001). Furthermore, we showed that at all time points, the ketamine-midazolam group had a higher MARS than the ketamine-propofol group. This novel finding in our study could indicate that the five MARS indices (respiration, activity, level of consciousness, circulation and oxygen saturation) were more stable in the ketamine-midazolam group than the ketamin-propofol group during PACU admission. Upon entering the PACU, a greater drop in blood pressure was seen in the ketamine-propofol group than the other two groups. This may be due to the vasodilatory effects of propofol or the timing of its injection.

Another key finding of our study was that the recovery time was significantly longer in the ketamine-propofol group relative to the other two groups (*P* = 0.008). This effect can be due to the concentration of propofol in the ketamin-propofol composition as well as its bolus injection at the end of the operation. In other studies, it was found that due to the counterbalance effect of drugs on each other, ketamin-propofol did not increase the recovery period compared with ketamine. However, as far as we know, no such comparison has been made between ketamin-propofol and ketamine-midazolam. As in this study the length of stay of the patient in recovery was shorter in the ketamine-propofol group and, on the other hand, the length of hospital stay was longer compared to the ketamine-midazolam group, perhaps the benefit and effectiveness of ketamine-midazolam is outweighed compared to ketamin-propofol. However, it should be noted that the optimal mixture and dosage of ketamine and propofol should be further investigated as in previous studies it was stated that a higher concentration of propofol (ketamine-propofol ratio of 1:3) could reduce not only the unwanted side effects (e.g. nausea) but also the recovery time (26–27). In our study, the ketamine-propofol ratio was 1:2.

Various factors play a role in causing EA, including pre-school age, the pressure of a urinary catheter, difficult parental separation behavior, the anaesthesia method, the presence of a tracheal tube, post-operative pain, the surgical procedure, anxiety and the patient’s personality (24, 28–29).

In our study, the pain score in the ketamin group was higher than the other two groups at all times. Upon entering the PACU and 20 min later, there was no significant difference in terms of pain between the two groups of ketamine-propofol and ketamine-midazolam, though at 5 min and 10 min after PACU admission, the pain score was significantly lower in the ketamine-propofol group than the two other groups (*P* < 0.001). Previous studies have emphasised the importance of effective post-operative pain management, such that several protocols have been described to control post-operative pain (9, 30–32). Post-operative pain affects various factors such as delirium and agitation; the study of González-Cardenas et al. (33) showed a direct relationship between delirium and pain. Pain goes through the nociceptors to the spinal cord and then to the thalamus, leading to the activation of the hypothalamic-pituitary-adrenal axis, which releases large amounts of cortisol-epinephrine and norepinephrine. This increase in epinephrine and norepinephrine can cause diaphoresis and anxiety in patients, which worsens emotional responses and agitation (34).

The main limitation in this study was the lack of sufficient information about different concentrations of propofol in ketamin-propofol and also the time of injection of drugs. In future studies, it is suggested that different doses of propofol in ketamin-propofol should be compared with the midazolam-propofol group to determine the best dose and time of injection. On the other hand, although the RASS in the ketamin-propofol group was lower than the other two groups at all times, due to the low MARS index in this group compared to the ketamin-midazolam group, further studies with larger sample sizes should be performed.

## Conclusion

In this study, it was observed that upon PACU admission, the RASS of patients in the ketamin-midazolam and ketamin-propofol groups was significantly lower than the ketamin group. Furthermore, during the entire recovery period, the ketamin-midazolam group had greater haemodynamic stability and MARS values than the other groups without delayed awakening.

## Figures and Tables

**Figure 2:**
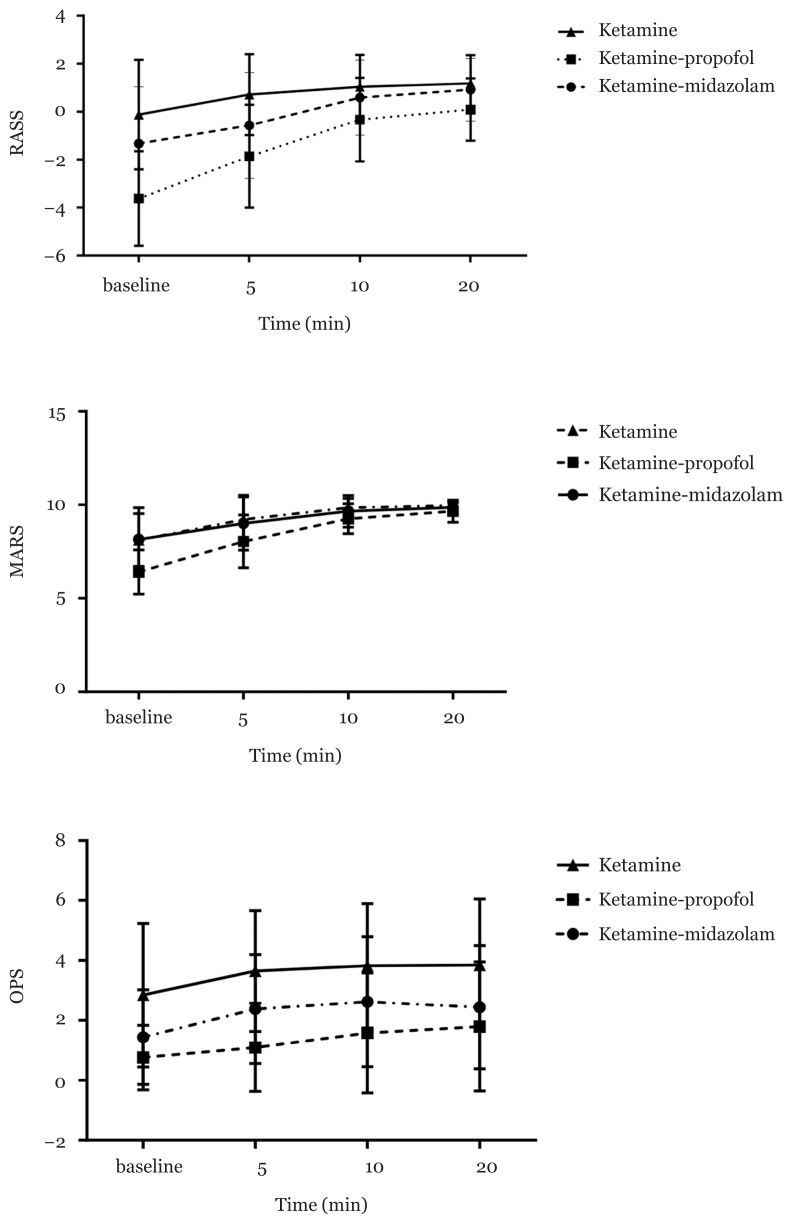
RASS, MARS and OPS in three groups during time

**Table 1:** Demographic variables among the three groups of the study

Groups		Ketamine	Ketamine-propofol	Ketamine-midazolam	*P*-value
Sex	Female	19 (38)	20 (40)	28 (56)	0.140
	Male	31 (62)	30 (60)	22 (44)	
Age (years old)	≤ 10	36 (72)	37 (74)	45 (90)	0.055
	10–15	14 (28)	13 (26)	5 (10)	

Note: Data are reported as *n* (%) and tested by Chi-squared test

**Table 2:** Comparisons of clinical variables among the three study groups

Group	Ketamine	Ketamine-propofol	Ketamine-midazolam	*P*-value
**RASS**				
Arrival to the recovery room	1 (−1, 2)	−5 (−5, −2.75)	−1 (−3, 0.5)	< 0.001#
5 min	1 (0, 2)	−1 (−4, 0)	0 (−1.5, 1)	< 0.001#
10 min	1 (0, 2)	0 (−1, 0.75)**	0 (0, 1)	< 0.001
20 min	1 (0, 2)	0 (0, 1)**	1 (0, 1)	< 0.001
**MARS**				
Arrival to the recovery room	8 (7, 9)	6 (6, 7)**	8.5 (6.75, 10)	< 0.001
5 min	10 (9, 10)	8 (7, 9)**	10 (8, 10)	< 0.001
10 min	10 (10, 10)	9 (9, 10)**	10 (10, 10)	< 0.001
20 min	10 (10, 10)	10 (9, 10)**	10 (10, 10)	< 0.001
**OPS**				
Arrival to the recovery room	3 (1, 4)*	0 (0, 1)	1.5 (0, 2)	< 0.001
5 min	4 (3, 5)	0 (0, 2)	2 (1, 3)	< 0.001#
10 min	4 (3, 5)	1 (0, 3)	3 (0.75, 4)	< 0.001#
20 min	4 (3, 5)*	1 (0, 3)	2 (0, 4)	< 0.001
Recovery time				
Time (min)	38.54 ± 5.83	41.48 ± 4.94**	38.76 ± 4.72	0.008

Notes: Data are reported as median (IQR) and tested by the Kruskal-Wallis test with Dunn’s test as post-hoc test and recovery time reported as mean ± SD and tested by the ANOVA test with Tukey test as post-hoc test;

# All groups has significant differences from each other in the pairwise comparison;

* Ketamine group has significant differences with other groups;

** Ketamine-propofol group has significant differences with other groups
